# IWATE criteria are associated with perioperative outcomes in robotic hepatectomy: a retrospective review of 225 resections

**DOI:** 10.1007/s00464-021-08345-w

**Published:** 2021-02-19

**Authors:** Kevin P. Labadie, David J. Droullard, Alex W. Lois, Sara K. Daniel, Kathryn E. McNevin, Jaqueline Valdez Gonzalez, Yongwoo D. Seo, Kevin M. Sullivan, Kyle S. Bilodeau, Lindsay K. Dickerson, Alan F. Utria, John Calhoun, Venu G. Pillarisetty, Jonathan G. Sham, Raymond S. Yeung, James O. Park

**Affiliations:** 1 Department of Surgery, University of Washington School of Medicine, 1959 NE Pacific Street, Health Sciences Bldg. Room BB-442, Box 356410, Seattle, WA 98195 USA; 2 Center for Advanced Minimally Invasive Liver Oncologic Therapies (CAMILOT), University of Washington, Seattle, WA 98195 USA; 3 Hepatobiliary Surgical Oncology, Department of Surgery, University of Washington Medical Center, 1959 NE Pacific Street, Health Sciences Bldg. Room BB-442, Box 356410, Seattle, WA 98195-6410 USA

**Keywords:** Robotic hepatectomy, Minimally invasive surgery, IWATE criteria, Robotic surgery

## Abstract

**Background:**

Robotic hepatectomy (RH) is increasingly utilized for minor and major liver resections. The IWATE criteria were developed to classify minimally invasive liver resections by difficulty. The objective of this study was to apply the IWATE criteria in RH and to describe perioperative and oncologic outcomes of RH over the last decade at our institution.

**Methods:**

Perioperative and oncologic outcomes of patients who underwent RH between 2011 and 2019 were retrospectively collected. The difficulty level of each operation was assessed using the IWATE criteria, and outcomes were compared at each level. Univariate linear regression was performed to characterize the relationship between IWATE criteria and perioperative outcomes (OR time, EBL, and LOS), and a multivariable model was also developed to address potential confounding by patient characteristics (age, sex, BMI, prior abdominal surgery, ASA class, and simultaneous non-hepatectomy operation).

**Results:**

Two hundred and twenty-five RH were performed. Median IWATE criteria for RH were 6 (IQR 5–9), with low, intermediate, advanced, and expert resections accounting for 23% (*n* = 51), 34% (*n* = 77), 32% (*n* = 72), and 11% (*n* = 25) of resections, respectively. The majority of resections were parenchymal-sparing approaches, including anatomic segmentectomies and non-anatomic partial resections. 30-day complication rate was 14%, conversion to open surgery occurred in 9 patients (4%), and there were no deaths within 30 days postoperatively. In the univariate linear regression analysis, IWATE criteria were positively associated with OR time, EBL, and LOS. In the multivariable model, IWATE criteria were independently associated with greater OR time, EBL, and LOS. Two-year overall survival for hepatocellular carcinoma and intrahepatic cholangiocarcinoma was 94% and 50%, respectively.

**Conclusion:**

In conclusion, the IWATE criteria are associated with surgical outcomes after RH. This series highlights the utility of RH for difficult hepatic resections, particularly parenchymal-sparing resections in the posterosuperior sector, extending the indication of minimally invasive hepatectomy in experienced hands and potentially offering select patients an alternative to open hepatectomy or other less definitive liver-directed treatment options.

Minimally invasive approaches for liver resection are increasingly utilized including laparoscopic and robot-assisted laparoscopic techniques, or robotic hepatectomy (RH). Over the last two decades, numerous single and multi-institutional case series report outcomes of RH, demonstrating comparable outcomes to standard laparoscopic and open hepatectomy [1–5]. Initially reserved for minor resections in the lateral sector, accumulated experience, technological advancements, and improvements in perioperative care have enabled RH for complex hepatectomy in many high-volume centers [6–9]. RH has been suggested to be advantageous to standard laparoscopic hepatectomy for certain resections, such as resection of posterosuperior hepatic segments; however, prospective data are lacking and comparing techniques in liver resection is limited by challenges in controlling for the extent and difficulty of resection [10–12].

To better control for these factors, several hepatectomy difficulty scoring systems have been developed based on patient and tumor factors. The first difficulty scoring system was developed by Ban et al. and was validated for RH[4, 13]. However, there were several limitations to the initial iteration, including omission of resections in segment 1, the inability to distinguish between a tumor in segment 4A or 4B or account for hand-assisted or hybrid approaches. This prompted its revision to the IWATE criteria in 2014, composed of a 4-level classification system accounting for the tumor location, its size, and proximity to a major hepatic vessel, the extent of liver resection, the utilization of a hybrid technique, and the patient’s liver function[14].

The aim of this study was to apply the IWATE criteria in RH and to characterize its association with perioperative outcomes, while reporting our initial institutional experience with RH over the last decade.

## Methods

### Study design and population

We performed a retrospective cohort study of all patients who underwent RH for primary hepatobiliary (PHB), metastatic, and benign liver tumors between 2011 and 2019 at the University of Washington Medical Center. Primary hepatobiliary (PHB) malignancies included hepatocellular carcinoma (HCC), intrahepatic cholangiocarcinoma (ICC), and gallbladder carcinoma (GBC). Diagnosis and treatment plans were made based on established criteria and guidelines from the National Comprehensive Cancer Network and other professional societies. All patients were evaluated by a multidisciplinary care team including experts from surgical, radiation, and medical oncology; transplant surgery and hepatology; diagnostic and interventional radiology; and pathology. The study protocol was approved by the Institutional Review Board and Human Subjects Research Division at the University of Washington.

### Operative technique and definitions

Liver segments were identified based on Couinaud’s segmental anatomic classification [15]. Anatomic resections were defined based on the Brisbane 2000 Terminology of Liver Anatomy and Resections [16]. Major liver resections were defined by resection of 3 or more adjoining Couinaud’s segments. The term “parenchymal-sparing” encompassed anatomic segmentectomy and non-anatomic partial resection.

All patients underwent RH using the da Vinci Surgical System™ (Intuitive Surgical, Inc, Sunnyvale, CA) *Xi* or *Si* models. Both extrahepatic and intrahepatic control are used and selected based on patient’s anatomy. Pringle maneuver was rarely used, and portal pedicles were controlled by extra or intrahepatic approach. Parenchymal transection was performed mainly with a Harmonic™ scalpel, with vascular control using Hem-o-lok™ clips or sutures, under ultrasound guidance using a drop-in probe. ICG fluorescence was occasionally used to identify planes of parenchymal resection. Operations were converted to an open approach when deemed necessary by the operating surgeon for management of prohibitive adhesive disease, hemorrhage, or failure to make timely progress. The primary robotic surgeons include authors JOP, RSY, VGP, and JGS. All cases were with a resident at bedside and rarely a second attending surgeon assisted at bedside or at surgeon console.

### Collected data

Patient demographics and perioperative data for all RH patients were collected, while oncologic outcomes were collected for patients with PHB malignancies with at least 6 months of follow-up. Patient demographics and clinical characteristics included age, gender, body mass index (BMI), ASA class, prior abdominal operation, and presence of moderate or severe steatosis or cirrhosis identified on surgical pathology. Perioperative data including the number and location of hepatic segments resected, operative time, estimated blood loss (EBL), operative blood products transfused, 30-day complications, conversion to an open operation, and length of hospital stay were collected. Operations that included concomitant surgical procedures, e.g., concurrent colectomy, were excluded from operative time, EBL, blood transfusion, and length of stay univariate analyses but included in multivariable model. The operation type, number and location of hepatic segments resected, the tumor size, proximity to major vasculature, and patient’s Childs Pugh score were collected, and the IWATE criteria were tabulated as described [14]. Segmentectomy was defined as a complete resection of an area supplied by a 3^rd^ order branch of the portal vein [17, 18]. When a tumor involved more than one Couinaud segment, the segment which was more involved by tumor was used for tabulation. The IWATE criteria were utilized to categorize the RH as a low- (criteria 0–3), intermediate- (criteria 4–6), advanced- (criteria 7–9), or expert-level (criteria 10–12) resection for analysis.

### Oncologic outcomes

Tumor characteristics including histologic subtype, tumor diameter, and resection margin status were collected from pathology reports for all resections. Oncologic outcomes, including disease-free survival and overall survival, were examined for PHB malignancies in patients with greater than 6 months of follow-up. Deaths were identified by EMR documentation, and disease recurrence was defined as documented tumor recurrence on surveillance computed tomography or magnetic resonance imaging. Based on these definitions, for patients with at least 2 years of follow-up care documented in the EMR, we calculated disease-free survival and overall survival at this time point. Assessing oncologic outcomes after resection of metastatic tumors was outside the scope of this work, as characteristics of the primary tumor and its systemic treatment more strongly influence survival than resection approach, and these data were not available. In addition, metastatic lesions are not routinely surveilled in our liver tumor clinic, and therefore, follow-up was limited.

### Statistical analysis

Categorical variables were expressed as frequency and percentages and analyzed using Chi-square or Fisher’s exact tests where appropriate. Continuous variables were expressed as medians and means and compared by Student’s t test or Mann–Whitney test depending on distribution of variables. To assess the relationship between IWATE score and OR time, EBL, and LOS, we first performed univariate linear regression with robust standard errors. To control for potential confounding by patient characteristics (age, sex, obesity, ASA class, prior abdominal operation, and simultaneous non-hepatectomy operation), we developed multivariable linear regression models using robust standard errors. Multivariable logistic regression was also performed to evaluate the association between IWATE criteria and 30-day postoperative complications. Results were considered significant with a *p* value < 0.05. Linear regression analysis was performed using Stata v. 16.0 (Stata Corp, College Station, TX, USA), categorical and continuous variables were compared in PRISM.

## Results

### Perioperative outcomes

Two hundred and twenty-five RH were performed at our institution during the study period with patient and resection characteristics as listed in Table 1. The majority of resections were parenchymal-sparing approaches, including anatomic segmentectomies and non-anatomic partial resections. The median IWATE criteria for RH were 6 (IQR 5–9), with low, intermediate, advanced, and expert resections accounting for 23% (*n* = 51), 34% (*n* = 77), 32% (*n* = 72), and 11% (*n* = 25) of resections, respectively (Table 2).

**Table 1:** Clinical characteristics, patient demographics, and resection specifics

Characteristics	RH (*n* = 225)
Age (yr), median (range)	56 (21–85)
Gender, female, *n* (%)	110 (49)
Body mass index, median (range)	26 (17–67)
Prior abdominal surgery, *n* (%)	140 (62)
ASA classification, *n* (%)	
2	64 (28)
3	144 (64)
4	9 (1)
Presence of moderate steatosis, *n* (%)	52 (23)
Indication, *n* (%)	
PHB Malignancy	92 (41)
HCC	68 (30)
ICC	17 (8)
GBC	7 (3)
Metastatic disease	98 (44)
Benign/other	34 (15)
Major resection, *n* (%)	57 (25%)
Type of resection, *n* (%)	
Anatomic resection	148 (66)
Left-lateral sectionectomy	21 (6)
Left hepatectomy	9 (4)
Right hepatectomy	15 (7)
Extended left lobectomy	1 (< 1)
Extended right lobectomy	2 (< 1)
Segmentectomy, *n* (%)	100 (43)
Non-anatomic resection, *n* (%)	77 (34)
Tumor diameter (cm), mean ± SD, maximum diameter	3.6 ± 2.5, 15
Hepatic segments involved by resected tumor (*n*)	
1	8
2	55
3	77
4A	29
4B	48
5	81
6	72
7	51
8	51

**Table 2:** Operative characteristics and perioperative outcomes by IWATE criteria

IWATE criteria	Low	Intermediate	Advanced	Expert
Number of resections	51	77	72	25
Partial resection, *n*	52	25	0	0
Left-lateral sectionectomy, *n*	0	21	1	0
Segmentectomy, *n*	0	30	57	13
Sectionectomy and more	0	0	14	12
Estimated blood loss (mL), median (IQR)	100 (50–150)	200 (80–400)	338 (184–863)	800 (300–1600)
Operative time (min), median (IQR)	216 (159–272)	230 (188–296)	330 (260–390)	400 (296–470)
Length of hospital stay (day), median (IQR)	2 (2–3)	3 (2–3)	3 (2–4)	4 (2.25–4.75)
Conversion to open surgery, *n* (%)	0	2 (1)	3 (4)	4 (15)
Complication rate (%)	14	10	14	28
Clavien-Dindo Grade 1, *n*	1	2	1	0
Clavien-Dindo Grade 2, *n*	4	5	8	4
Clavien-Dindo Grade 3, *n*	1	1	1	3
Clavien-Dindo Grade 4, *n*	1	0	0	0
R0 resection (%)	93	85	86	80

Conversion to open hepatectomy occurred in 9 patients (4%), seven of them during advanced- or expert-level cases (IWATE criteria ≥ 7). Four conversions occurred for hemorrhage control (IWATE criteria: 1 intermediate, 2 advanced, 1 expert), two for extensive adhesions (IWATE criteria: 2 expert), two for failure to progress (IWATE criteria: 1 advanced, 1 expert), and one for diaphragmatic involvement (IWATE criteria: 1 intermediate). Postoperative complications occurred within the first 30 days in 32 patients (14%) with most common being superficial and deep surgical site infection (*n* = 7, 3%), atrial fibrillation (*n* = 4, 2%), and pulmonary embolism (*n* = 3, 1%). The majority (*n* = 17, 53%) of complications within 30-days occurred after advanced or expert-level resections (IWATE criteria ≥ 7). The median length of stay was 3 days (IQR 2–4), and there were no deaths within 30 days postoperatively.

Of the 25 RH categorized as expert-level resections, 10 right hepatectomies (rH), 2 extended right hepatectomies (erH), and 13 posterosuperior segmentectomies (psS) were performed. They were most commonly performed for metastatic tumors (*n* = 12) and HCC (*n* = 9), and the majority occurred in the second half of the study period (*n* = 16). Sixteen percent (*n* = 4) of expert-level resections were converted to open surgery (2 for adhesive disease for erH, 1 for hemorrhage control for psS, 1 for failure to progress for psS). Twenty eight percent (*n* = 7) had postoperative complications (seroma, biloma, small bowel obstruction, pneumothorax, aspiration pneumonia, intra-abdominal abscess requiring percutaneous drainage). The expert-level segmentectomies were performed for large tumors (mean tumor size 5.1 cm) in segment 4A, 7, and 8 with median EBL of 1320 mL, median OR time of 406 min, complication rate of 23%, and R0 resection rate of 70%.

**Fig. 1:**
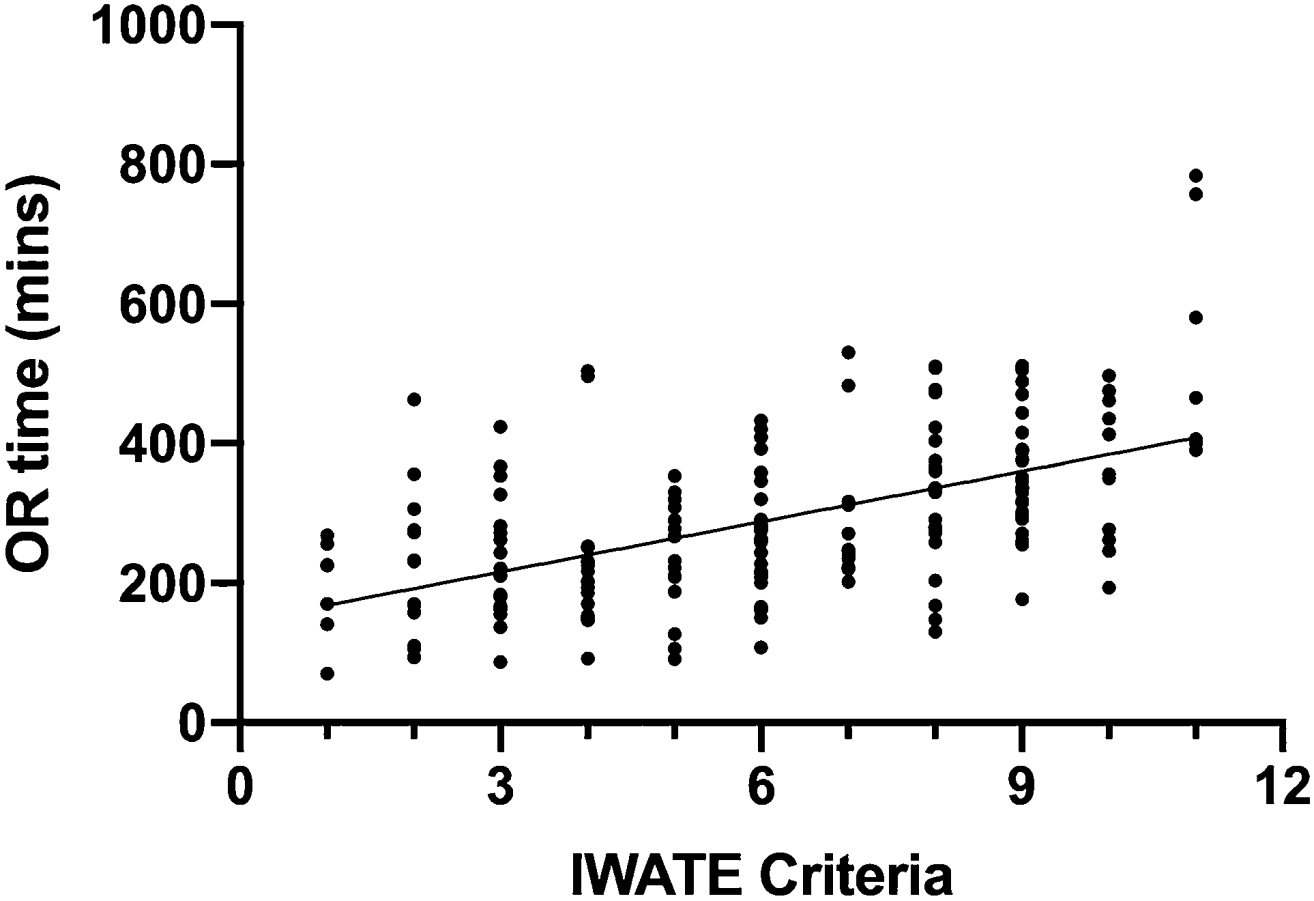
Correlation of IWATE criteria with operative time (*R*^2^ = 0.30, *p* < 0.001)

**Fig. 2:**
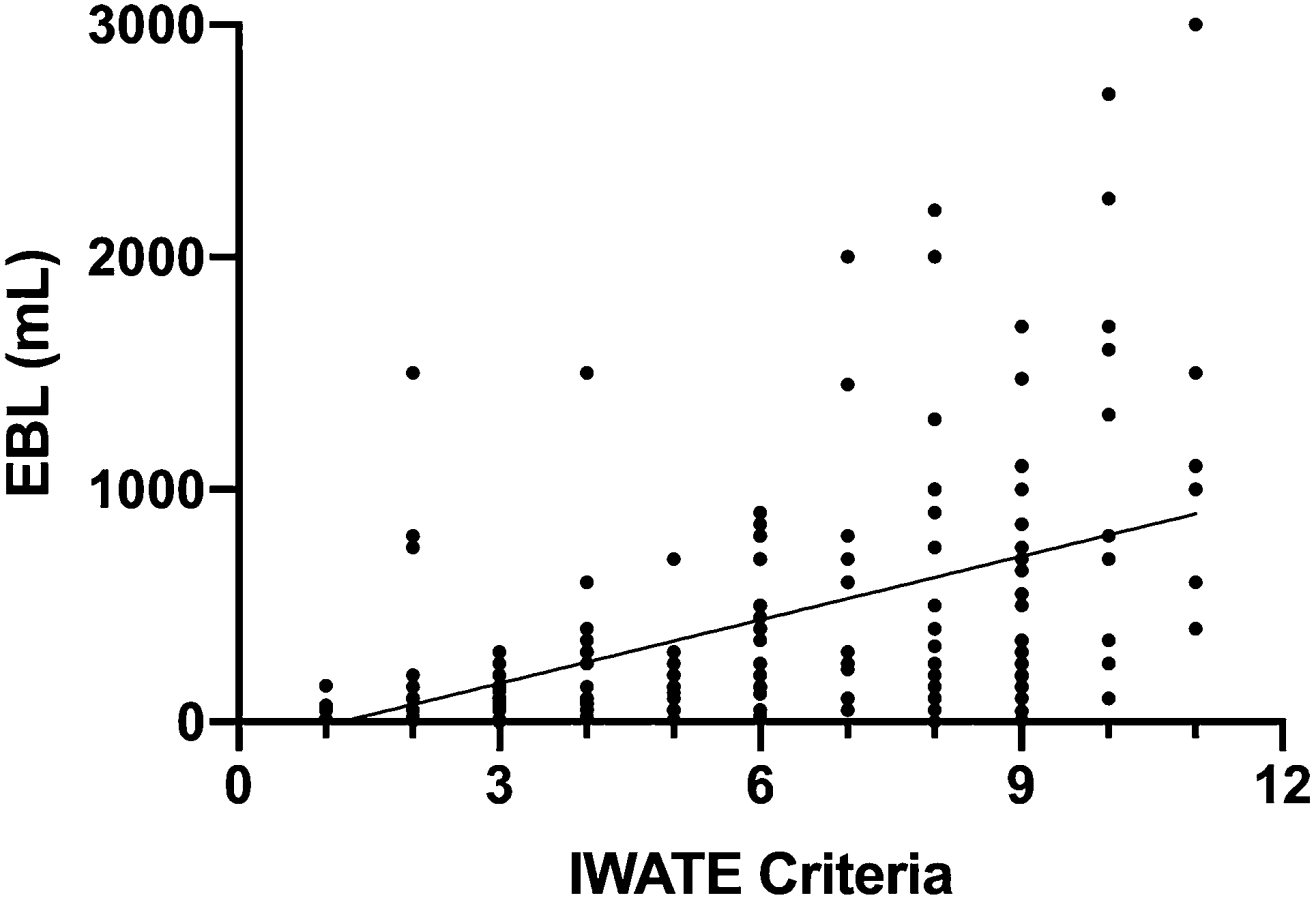
Correlation of IWATE criteria with estimated intraoperative blood loss (*R*^2^ = 0.22, *p* < 0.001)

**Fig. 3:**
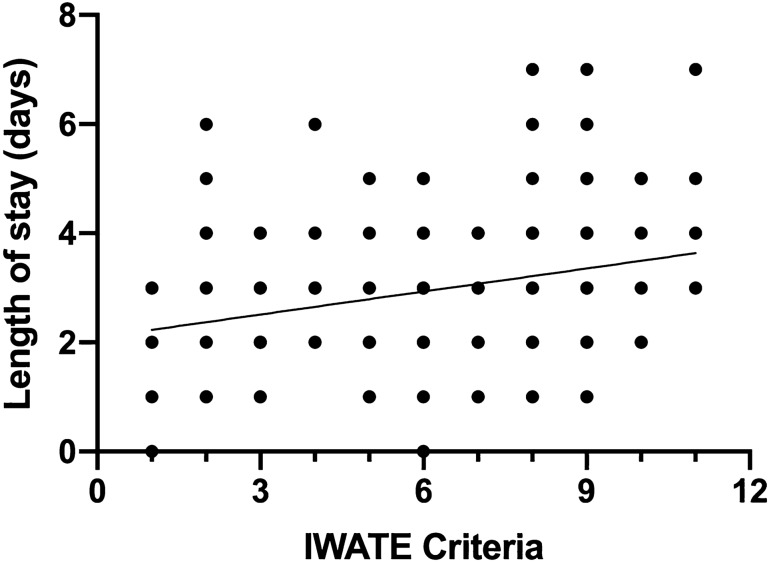
Correlation of IWATE criteria with postoperative length of hospital stay (*R*^2^ = 0.08, *p* < 0.001)

In the univariate linear regression analysis, IWATE criteria were positively associated with OR time, EBL, and LOS (Figs. 1, 2, 3). In the multivariable model, IWATE criteria were independently associated with greater OR time, EBL, and LOS (Tables 3, 4, 5). We estimate that each additional IWATE criterion was associated with a 24 min greater mean OR time (95% CI 18–30 min, *p* < 0.0005), 110 cc greater mean EBL (95% CI 81–139 cc, *p* < 0.0005), and 0.22 days greater mean LOS (95% CI 0.13, 0.31, *p* < 0.0005). Combined cases were independently associated with greater OR time and length of stay but not EBL; none of the other patient characteristics were independently associated with these outcomes. IWATE criteria were not associated with an increase in 30-day complication rate (OR 1.10, 95% CI 0.95–1.27, *p* = 0.19).

**Table 3:** Relationship between IWATE criteria and OR Time (minutes)

Independent Variable	Coefficient	95% CI		*p*
IWATE Criteria	23.67	17.78	29.56	< 0.0005
Age	− 1.22	− 2.49	0.06	0.061
Female sex	14.28	− 18.94	47.51	0.398
Obesity	2.04	− 33.26	37.33	0.91
Prior Abdominal Surgery	7.63	− 26.35	41.61	0.659
ASA Class	12.54	− 15.28	40.36	0.375
Combined Case	185.26	142.90	227.63	< 0.0005
Constant	171.24	67.92	274.55	0.001

*R*^2^ = 0.387

**Table 4:** Relationship between IWATE criteria and EBL (cc)

Independent variable	Coefficient	95% CI		*p*
IWATE Criteria	109.57	80.58	138.55	< 0.0005
Age	− 3.08	− 9.40	3.23	0.34
Female sex	− 184.62	− 348.61	− 20.62	0.03
Obesity	65.41	− 108.31	239.12	0.46
Prior Abdominal Surgery	59.43	− 108.47	227.33	0.49
ASA class	47.09	− 88.27	182.45	0.49
Combined case	20.71	− 183.24	224.66	0.84
Constant	− 80.65	− 586.20	424.90	0.75

*R*^2^ = 0.231

**Table 5:** Relationship between IWATE criteria and LOS (days)

Independent variable	Coefficient	95% CI		*p*
IWATE Criteria	0.219	0.131	0.307	< 0.0005
Age	0.011	− 0.008	0.031	0.244
Female sex	− 0.027	− 0.527	0.472	0.915
Obesity	0.026	− 0.502	0.553	0.924
Prior abdominal surgery	0.173	− 0.339	0.684	0.506
ASA class	0.340	− 0.072	0.752	0.105
Combined case	1.825	1.202	2.448	< 0.0005
Constant	0.091	− 1.447	1.629	0.907

*R*^2^ = 0.203

### Oncologic outcomes

Eighty-four patients who underwent RH for a PHB malignancy (63 HCC, 14 ICC, 7 GBC) had sufficient follow-up data to be included in the oncologic analysis (Table 6). Median follow-up was 22 months (range 6–87). R0 resection rate for HCC, ICC, and GBC were 91%, 93%, and 71%, respectively. 2-year local recurrence rates for HCC and ICC were 14%, 0%, and 20%, respectively. 2-year disease-free survival for HCC, ICC, and GBC was 55%, 17%, and 80%, respectively. 2-year overall survival for HCC, ICC, and GBC was 94%, 50%, and 100%, respectively.

**Table 6:** Oncologic outcomes

	HCC (n = 63)	ICC (n = 14)	GBC (n = 7)
Largest tumor diameter (cm), median (range)	4.1 (1–12)	4.1 (2–7)	2.2 (0.1–6)
R0 resection, n (%)	57 (91)	13 (93)	5 (71)
1-year local recurrence rate (%)	8	0	20%
2-year local recurrence rate (%)	14	0	20%
1-year disease-free survival (%)	75	75	80
2-year disease-free survival (%)	55	17	80
1-year overall survival (%)	96	75	100
2-year overall survival (%)	94	50	100

## Discussion

We describe our institutional experience of 225 RH, which to our knowledge is the largest reported cohort study to date and characterize the association between IWATE criteria and outcomes after RH. In this cohort, RH is shown to be safe and feasible, with an overall complication rate of 14% and a 4% open conversion rate. Oncologic outcomes for PHB malignancies were comparable to other published series with 2-year local recurrence rate of 14%, 55% 2-year disease-free survival rate, and a 94% 2-year overall survival rate for HCC [19].

The IWATE criteria were developed in 2014 during the Second International Consensus Conference on Laparoscopic Liver Resections held in Morioka, Japan, in the IWATE prefecture [14]. The criteria made minor modifications to the original Ban laparoscopic difficulty score [13], including segment 1 resections, differentiating segment 4A from 4B, and accounting for the hand-assist laparoscopic or hybrid method. The IWATE criteria have been extensively validated in standard laparoscopic hepatectomy [20, 21], and only recently applied to RH [22]. In our study, we found the criteria be associated with perioperative variables often observed with more challenging resections, including higher EBL, OR time, and LOS on both univariate and multivariable analyses. This classification system may represent an important tool for robotic liver surgeons to predict the difficulty of RH according to preoperative variables and to safely select cases for RH by skill level.

We observed a significant proportion of RH to be used for higher difficulty resections based on IWATE criteria, with 43% of RH performed for advanced- or expert-level resections during our initial institutional experience. In our series, we performed higher numbers of anatomic segmentectomies (43%) and lower major anatomic hepatectomies (13%) compared to other published minimally invasive series [21]. This reflects our institutional approach to favor parenchymal-sparing approaches when possible. RH for these resections showed higher EBL, OR time, and complication rate compared to resections in other segments but achieved comparable R0 resection rates and LOS.

These data support the findings of several groups that have reported a unique advantage of the robotic surgical platform in performing parenchymal-sparing liver resections of the posterosuperior segments that are difficult to access via standard laparoscopy [10, 12, 23–25]. In contrast to performing a right hepatectomy laparoscopically for tumors located in the posterior segments, a parenchymal-sparing robotic approach to these segments is advantageous for maximally preserving future liver remnant. Sparing parenchyma is becoming increasingly important in cancer patients, particularly those with colorectal cancer metastases, who are living longer with more effective and hepatotoxic systemic chemotherapy regimens and often benefit from resection of intrahepatic recurrence [26, 27]. At our institution, the safe and effective adoption of RH for these difficult segmental resections supports extending the indication for RH and establishes it as a minimally invasive parenchymal-sparing alternative to major resection or conventional open minor resection when technically feasible. Examination and comparison of outcomes after RH with open and standard laparoscopic resection using propensity score matching at our institution are ongoing.

This study has several important limitations. First, this is a single center study limiting its generalizability and introducing institutional and surgeon bias for robotic technique. Furthermore, as a retrospective series using chart review, data collection was limited to information available in the electronic medical record. In regard to oncologic outcomes, as the University of Washington Medical Center is a tertiary care referral health system that serves the Washington, Wyoming, Alaska, Montana, and Idaho (WWAMI) region, many patients in this series who came to our center for their initial consultation and subsequent operation were lost to follow-up when they returned to their local oncology providers for ongoing care; therefore, oncologic outcomes such as recurrence and death were censored. Given that our multidisciplinary clinic provides comprehensive specialty care for liver tumors and requests follow-up information on surveillance imaging performed by our referring providers, we do expect that most cases of recurrence were captured in our review of outside records; conversely, patients who enjoyed a good response likely had no reason to follow-up with our clinic, and providers were more likely to contact us back regarding patients who had developed recurrence than those that had not. This reporting bias may negatively influence our results to show worse oncologic outcomes than truly occurred.

In conclusion, in our series of 225 RH, we characterized the association between the IWATE criteria and important operative outcomes, highlighting its utility in predicting resection difficulty and assisting in appropriate patient selection for RH. Our series also highlight the utility of RH for difficult hepatic resections, particularly parenchymal-sparing resections in the posterosuperior segments, extending the indication of minimally invasive hepatectomy in experienced hands and potentially offering select patients an alternative to open hepatectomy or less definitive alternative liver-directed therapies.
